# Supplementary material for: Learning curve and surgical outcome of robotic assisted colorectal surgery with ERAS program
Source: Sci Rep. 2022 Nov 29;12:20566. doi: 10.1038/s41598-022-24665-w (PMC9709162; doi:10.1038/s41598-022-24665-w)
Supplement: Supplementary file 1 — Supplementary Information 1. [file 41598_2022_24665_MOESM1_ESM.zip › LSC Raw data-Ñ[▒K/147/N_ñw╝╨░O▒Kñσ.pdf]

W92 / W92

臺中榮民總醫院
手術室護理記錄

[redacted]

基本資料

- 室別： 開刀房-40室，手術科別： CRS，手術類別： 常規，日期： 2022/01/03
- 進入時間： 1410 ； 麻醉開始時間： 1415 ； Time Out時間： 1525 ； 劃刀時間： 1526 ； 手術結束時間： 1850 送出時間： 1905 ；
- 麻醉方式： GE
  診斷：RS COLON CA WITH LEFT ADENEXA LESION
  手術名稱：Laparoscopic low anterior resection of rectum
  手術局部用藥：NIL
  術前預防性抗生素：
  - 抗生素名稱一： Veterin 1GM IVD/AR
  - 時間： 1500
  - 上次簽章: 陳昱穎

  ，追加預防性抗生素：
- 手術醫師： 蔣鋒帆
- 刷手護士A ： 李宜芳，時間： 1410，接替刷手一： 李庭，時間： 1500，接替刷手二： 陳昱穎，時間： 1730
  流動護士A ： 黃仁慧，時間： 1410，接替流動一： 陳昱穎，時間： 1500，接替流動二： 卓汶珊，時間： 1700
- 更多接替及流動護士： 接替流動三： 李庭，時間： 1800

皮膚狀況

A-V Shunt： 無
術前： 完整
記錄者： 上次簽章: 黃仁慧
術後： 完整
記錄者： 李庭 上次簽章: 陳昱穎

手術準備

手術臥式 單一手術臥式

-
  - 手術臥式： 截石臥式
  - 使用附件： 溫毯 38 ℃，腳架
  - 皮膚準備： 10%Aq Betadine，Hibiscrub，2% Chlorhexidine
  - 電燒： 使用電燒: 電極片位置： 左背部，其它： 床上導電軟墊，右背部，其它： 床上導電軟墊，記錄者： 上次簽章: 黃仁慧

止血帶T： 無

植入物

- SURGIWRAP/PELVIC/壹/MAST/REF25202-01/LOT:54779
  ENDO GIA 60*4.1MM X2，CEEA 33X1 於COLON

，記錄者: 李庭

標本送檢

- 細菌培養 無
- 細胞學 無
- 冰凍切片 無
- 病理標本
  組織名稱一：RS COLON
  ，
  組織名稱二：DISTAL CUT END
  ，
  組織名稱三：IMA ROOT
  ，更多組織名稱
  -

- ○ 組織名稱四：UTERUS SURFACE
- 截肢 無

標本交付 標本： 3 袋，標本： 0 盒，標本： 1 瓶，細胞學： 0 管，Culture Tube： 0 管，被交付醫師： Fellow 楊曙榮，

- 記錄者： 李庭
- 時間： 1845

傷口術後敷料

傷口敷料： 傷口敷料： 無

- 請於圖上標示部位：

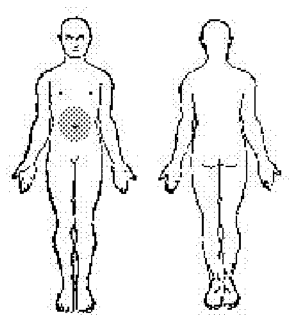

腹腔鏡切口五個

- 傷口敷料： mepore

導管

導管	天數	導管資訊	插入日期	插入時間	更換日期	拔除日期	拔除時間	拔除原因	預計拔除/更換	預計拔除/更換日期
Double Lumen CVC(1)	1	號碼:8 Fr; 部位:; 象限:RU; 註記:fix 15 cm;	2022/01/03	1450						
Foley(1)	1	號碼:16 Fr.; 部位:膀胱;	2022/01/03	1445						
A-line (1)	1	號碼:20 號; 部位:右手;	2022/01/03	1440						

用物計數

- 手術中出血量： MINIMAL c.c.
- 手術中尿量： 650 c.c.
-
  - ○ 其它： 無

用物計數： 多切口同時縫合

- 1.器械： 正確，2.縫針： 正確，3.刀片： 正確，4.紗布： 正確，5.腹部墊： 未使用，6.紗球： 未使用，7.PEANUTS ： 未使用，8.紗條： 未使用，9.絲帶： 未使用，10.針頭： 未使用，11.Vessel loop： 未使用，12.Boots： 未使用，13.Jelco： 未使用，14.棉條： 未使用，
  - ○ 第一次：
    - ▪ 刷手護士 ： 陳昱穎
    - ▪ 流動護士： 李庭 上次簽章: 陳昱穎
    - ▪ 時間： 1816

  ,
  - ○ 第二次：
    - ▪ 刷手護士 ： 陳昱穎
    - ▪ 流動護士： 李庭 上次簽章: 陳昱穎
    - ▪ 時間： 1830

  ,
  - ○ 第三次：
    - ▪ 刷手護士 ： 陳昱穎
    - ▪ 流動護士： 李庭 上次簽章: 陳昱穎
    - ▪ 時間： 1840

備註

備註： NIL

- 記錄者： 上次簽章: 陳昱穎
- 時間： 1600

填寫護理人員 李庭

正式摘要

		W92 / W92			

doctorRank:B
